# Holter-determined arrhythmias in young elite athletes with suspected risk: Insights from a 20-year experience

**DOI:** 10.3389/fcvm.2022.896148

**Published:** 2022-07-22

**Authors:** Araceli Boraita, María-Eugenia Heras, Pedro L. Valenzuela, Leonel Diaz-Gonzalez, Francisco Morales-Acuna, María Alcocer-Ayuga, Sonia Bartolomé-Mateos, Alejandro Santos-Lozano, Alejandro Lucia

**Affiliations:** ^1^Department of Cardiology, Sports Medicine Center, Consejo Superior de Deportes, Madrid, Spain; ^2^Research Institute of the Hospital 12 de Octubre (“imas12”, PaHerg group), Madrid, Spain; ^3^Department of Cardiology, CEMTRO Clinic, Madrid, Spain; ^4^Department of Cardiology, La Paz Hospital, Madrid, Spain; ^5^Especialidad en Medicina del Deporte y la Actividad Física, Facultad de Ciencias, Universidad Mayor, Santiago, Chile; ^6^Department of Health Sciences, i+HeALTH Research Group, European University Miguel de Cervantes, Valladolid, Spain; ^7^Faculty of Sport Sciences, Universidad Europea de Madrid, Madrid, Spain

**Keywords:** cardiac rhythm, ECG, sports, echocardiography, Holter monitoring

## Abstract

**Purpose:**

We assessed the occurrence of rhythm alterations in elite athletes with suspected risk using Holter monitoring, and the association of Holter-determined rhythm alterations with echocardiographic findings.

**Methods:**

A large cohort of Spanish elite athletes (*N* = 6,579, 34% female) underwent in-depth cardiological examination (including echocardiographic evaluation, and resting and exercise electrocardiogram [ECG]) between 01/02/1998 and 12/31/2018. Holter monitoring was performed in those reporting cardiovascular symptoms, with suspicion of cardiac structural abnormalities potentially associated with dangerous arrhythmias, or with resting/exercise ECG features prompting a closer examination. We assessed the occurrence of cardiac rhythm alterations, as well as the association between echocardiography-determined conditions and rhythm alterations.

**Results:**

Most athletes (*N* = 5925) did not show any sign/symptom related to arrhythmia (including normal resting and exercise/post-exercise ECG results) whereas 9.9% (*N* = 654; 28% female; median age, 24 years [interquartile range 19–28]; competition experience [mean ± SD] 10±6 years) met the criteria to undergo Holter monitoring. Among the latter, sinus bradycardia was the most common finding (present in 96% of cases), yet with a relatively low proportion of severe (<30 bpm) bradycardia (12% of endurance athletes during night-time). Premature atrial and ventricular beats were also common (61.9 and 39.4%, respectively) but sinus pauses ≥3 s, high-grade atrioventricular blocks, and atrial fibrillation/flutter were rare (<1%). Polymorphic premature ventricular contractions (PVC, 1.4%) and idioventricular rhythm (0.005%) were also rare. PVC couplets were relatively prevalent (10.7%), but complex ventricular arrhythmias were not frequent (PVC triplets: 1.8%; sustained ventricular tachycardia: 0.0%; and nonsustained ventricular tachycardia: 1.5%). On the other hand, no associations were found between arrhythmias (including their different morphologies) and major cardiac structural alterations (including mitral prolapse). However, an association was found between mild mitral regurgitation and supraventricular (odds ratio 2.61; 95% confidence interval 1.08–6.32) and ventricular (2.80; 1.15–6.78; *p* = 0.02) arrhythmias, as well as between mild or moderate mitral regurgitation and ventricular arrhythmias (2.49; 1.03–6.01).

**Conclusions:**

Irrespective of the sports discipline, “dangerous” ventricular arrhythmias are overall infrequent even among young elite athletes who require Holter monitoring due to the presence of symptoms or abnormal echocardiographic/ECG findings, and do not seem to be associated with underlying serious cardiac structural pathologies.

## Introduction

Elite athletes represent the top tier in competitive sports, and their long-term strenuous training regimes induce unique physiological adaptations, particularly at the cardiovascular level (1). This includes changes not only in cardiac dimensions (such as left ventricular hypertrophy) but also in electrical activity (mainly sinus bradycardia) (2, 3). In this regard, although regular moderate exercise confers protection against cardiac (including fatal) arrhythmias, principally by improved autonomic balance (4), concerns exist as to whether strenuous—especially endurance—exercise might have the opposite, nonphysiological effect, with elite athletes potentially at high risk of arrhythmias (5–7), including dangerous arrhythmias. Several studies have reported the prevalence of ventricular arrhythmias and sinus pauses in athletes of different training levels (8–11), but relatively scarce data are available in highly competitive (i.e., “elite”) athletes based on Holter monitoring (9, 11–16), the method that provides more information for detection of cardiac rhythm alterations than resting 12-lead electrocardiogram (ECG) recordings.

In the present study, we analyzed the occurrence of cardiac rhythm alterations in a large group of elite athletes who underwent Holter monitoring due to suspected risk. We also assessed the potential association between Holter-determined cardiac rhythm alterations and echocardiography-determined cardiac abnormalities. Our main hypothesis was that cardiac rhythm alterations are very infrequent in elite athletes.

## Materials and methods

### Study design and participants

The present study follows the “Strengthening the Reporting of Observational Studies in Epidemiology” (STROBE) guidelines. The study was conducted at the Cardiology Department of the ‘Consejo Superior de Deportes’ (CSD, Madrid, Spain). In this center, Spanish elite athletes of different disciplines who are part of the national team in their specialty and compete in major international events (e.g., Olympic Games, European or World championships) undergo in-depth cardiological evaluation (≥1 per year). The study was approved by the local Ethics Committee (IRB #1385226-1) and complies with the Declaration of Helsinki and its later amendments. Oral or written consent was obtained from all participants.

All cardiological evaluations performed between January 2nd, 1998 and December 31st, 2018 were retrospectively reviewed. During this period, each elite athlete underwent at least one complete evaluation that included medical history, physical examination, resting 12-lead resting ECG, and echocardiography evaluations (see below), and exercise testing for cardiorespiratory fitness (maximum oxygen uptake, VO_2_max) determination together with 12-lead ECG recordings, as detailed by us elsewhere (17, 18). Echocardiography evaluations were conducted using a Toshiba SSH-140A system (Toshiba Medical Systems, Tochigi, Japan) equipped with 2.5- and 3.75-MHz probes, or a Phillips Sonos 7500 system (Advance Diagnostics, Palo Alto, CA) equipped with a color, tissue Doppler, multifrequency 2-4 MHz transducer. All measurements were taken independently by two experienced sonographers (A.B. [>30-year experience] and M.E.H. [>15-year experience], 15 years working together), with all the evaluations ultimately supervised by the same researcher (AB). Ventricular and atrial dimensions were measured using two dimensional (2D)-guided M-mode imaging and 2D imaging, respectively, following American Society of Echocardiography recommendations (19). Left ventricular diastolic function was assessed by measuring transmitral flow rate (pulsed-wave Doppler, apical four-chamber view) and determining E and A wave velocities, also following ASE recommendations (20). All participants were assessed under resting conditions (i.e., during morning hours or early afternoon, after a rest period from the last exercise training session of at least 12 hours).

In addition, Holter monitoring was conducted in those athletes meeting one or more of the following criteria at any given time point during the aforementioned period: family history of sudden cardiac death and/or cardiomyopathy, reporting cardiovascular symptoms (palpitations at rest, syncope, dizziness, chest pain); reaching abnormally/unexpectedly high heart rates during training sessions; suspicion of cardiac structural abnormality potentially associated with an increased risk of dangerous arrhythmias (i.e., cardiomyopathies of any type); showing resting and/or exercise ECG features prompting a deeper examination (i.e., bradycardia <40 bpm in resting ECG or sinus pauses, atrioventricular [AV] and fascicular blocks); atrial fibrillation (AF) or flutter, Wolf-Parkinson-White syndrome, paroxysmal supraventricular tachycardia, frequent premature ventricular contractions (PVC) in resting (i.e., ≥ two in 10 s) or exercise/post-exercise ECG (isolated, couplets or triplets), or long QT in resting ECG. Holter monitoring ECG recording was performed continuously for 24 h (or 48 h in those athletes with long QT or suspected cardiomyopathy) by means of a 3-channel, 7-lead SEER Light recorder (GE Healthcare, Milwaukee, WI), using MCL1 to determine PVC morphology (i.e., left [LBBB] or right bundle branch block [RBBB]), III to determine PVC axis, and CM5 to corroborate PVC morphology and to assess the potential presence of ischemia. The totality of Holter data were analyzed by the same researcher that supervised all the echocardiographic evaluations (i.e., A.B.). using GE MARS PC Holter software (GE Healthcare). Each Holter record was subjected to beat-to-beat inspection for artifact removal and cardiac rhythm classification. Participants used a diary to register any symptoms and also their waking up and sleeping times. The following outcomes were determined for each evaluation: bradycardia, atrial rhythms (premature atrial beat, AF/flutter, wandering pacemaker, supraventricular tachycardia), junctional rhythms (premature junctional beat, idioventricular rhythm), ventricular rhythms (PVC, ventricular tachycardia), sinus pauses above 2 or 3 s, AV blocks (1st, 2nd, high-grade and 3rd degree, respectively), isolated PVC (1–99, 100–999, ≥1,000, and the different PVC morphologies).

### Statistical analysis

Data are presented as mean (standard deviation) for continuous variables (and as mean [interquartile range] for age) or as frequency (percentage) for categorical variables unless otherwise stated. In those athletes with more than one Holter evaluation, only data from the evaluation showing cardiac rhythm alterations was used (or alternatively the most recent evaluation in the case that no alterations were found). The normal distribution of the data was assessed with the Shapiro-Wilk test and confirmed by visual inspection. Unpaired Students' *t* test was used to compare the main characteristics of athletes undergoing or not, respectively, Holter evaluations. In those athletes undergoing Holter evaluation, a one-way analysis of variance was used to assess differences between types of sport (i.e., skill, power, mixed or endurance). The Greenhouse-Geisser correction factor was applied when the assumptions of sphericity were violated. When a significant group (i.e., “main type of sport discipline”) effect was found, the Bonferroni test was used *post hoc* for pairwise comparisons. The chi-squared test (or Fisher's exact test if >20% of the cells in the cross-table had an expected frequency <5) was used for the analysis of categorical variables. Logistic regression analysis was used to assess the potential association between arrhythmias and echocardiography-determined abnormalities. Analyses were conducted using SPSS 20.0 (IBM, Armonk, NY) and significance was set at *p* < 0.05.

## Results

A total of 19,662 in-depth cardiological evaluations were performed during the 20-year study period in 6,579 elite athletes, with each athlete undergoing on average three complete evaluations and 63% of athletes undergoing two or more evaluations. There were no missing data. The main characteristics of the study cohort were as follows: age median age 24 years (19–28), 34 % female, body mass index 22.6 ± 16.2 kg·m^−2^, training volume 19 ± 9 h/week, 8 ± 5 years in competition, and VO_2_max 53.7 ± 9.4 ml·kg^−1^·min^−1^.

**Figure 1:**
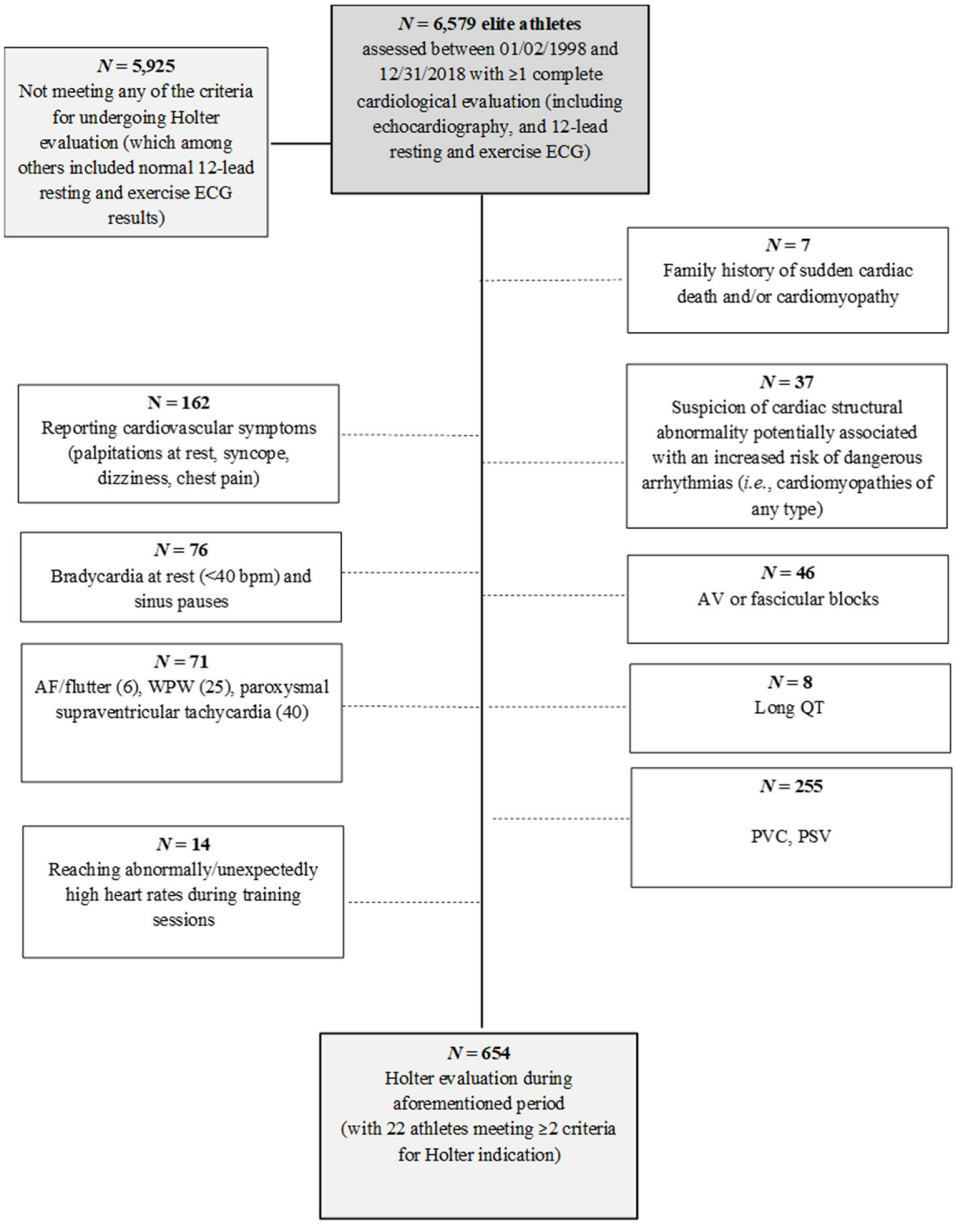
Participant flow chart.

Of the total sample, 654 athletes (9.9% of total, 28% female) underwent Holter monitoring at least once as part of their cardiological evaluation (see more details in Figure 1). Most of these athletes (81%) underwent a single Holter evaluation whereas 83 (13%) and 39 (6%) underwent two and three or more evaluations, respectively, with all evaluations including at least one hard training session. The main characteristics of the athletes who underwent or not Holter monitoring, respectively, are shown in Supplementary Material. The former group had a lower proportion of women, a higher proportion of endurance athletes, a younger age (with less years in elite competition), higher VO_2max_ values and resting ECG-determined heart rate, and overall larger cardiac dimensions.

The descriptive characteristics of study participants undergoing Holter evaluation attending to the main type of sport discipline are shown in Table 1. Concisely, resting ECG-determined heart rate as well as Holter-determined average day and night-time heart rate values ranged in the following order: skill > power > mixed > endurance. The opposite trend was found for VO_2_max levels.

**Table 1:** Demographics and training characteristics of the participants who underwent Holter monitoring.

	**Overall** ***N*** = **654**	**Skill** ***N*** = **43**	**Power** ***N*** = **135**	**Mixed** ***N*** = **179**	**Endurance** ***N*** = **297**	***p*** **-value**
Age (years)	24 (19–28)	21 (17–27)	22 (18–26)^d^	23 (18–28)	25 (20–29)^b^	0.018
Training regimen (hours/week)	19 ± 8	20 ± 11	19 ± 7^c^	16 ± 7^b^, ^d^	21 ± 8^c^	<0.001
Years in competition	10 ± 6	9 ± 4	9 ± 5	11 ± 7^d^	9 ± 6^c^	0.015
VO_2_max (ml/kg/min)	56.7 ± 10.1	48.2 ± 8.5^c^, ^d^	50.6 ± 9.1^d^	53.4 ± 7.1^a^, ^d^	62.3 ± 9.0 ^a^, ^b^, ^c^	<0.001
ECG heart rate (bpm)	56 ± 13	68 ± 14^b^, ^c^, ^d^	59 ± 13^a^, ^d^	57 ± 12 ^a^, ^d^	53 ± 12^a^, ^b^, ^c^	<0.001
**Holter heart rate,** daytime (bpm)^*^						
Average	74 ± 12	81 ± 13^c^, ^d^	77 ± 12^d^	75 ± 12^a^, ^d^	70 ± 11^a^, ^b^, ^c^	<0.001
Maximum	167 ± 23	165 ± 22	164 ± 24	169 ± 23	167 ± 23	0.287
Minimum	45 ± 8	50 ± 10^c^, ^d^	47 ± 8^d^	45 ± 7^a^, ^d^	43 ± 8^a^, ^b^, ^c^	<0.001
**Holter heart rate, night-time (bpm)**						
Average	52 ± 9	59 ± 11^c^, ^d^	55 ± 9^d^	53 ± 9^a^, ^d^	50 ± 8^a^, ^b^, ^c^	<0.001
Maximum	92 ± 14	98 ± 16^c^, ^d^	96 ± 12^d^	92 ± 15^a^, ^d^	90 ± 13^a^, ^b^	<0.001
Minimum	37 ± 7	42± 8^c^, ^d^	40 ± 6^c^, ^d^	37 ± 6^a^, ^b^, ^d^	36 ± 6^a^, ^b^, ^c^	<0.001

*Data are mean ± SD, except for age (median [intequartile range]). The main different types of sports in each discipline were [attending to recent classification (21)]: archery, motor driving, golf, jet skiing, motorcycling, and table tennis for “skill” disciplines; alpine skiing, artistic gymnastics, boxing, karate, snowboarding, track and field sprint/throwing/jumping events, weightlifting and wrestling for “power”; badminton, basketball, handball, field hockey, fencing, kickboxing, rugby, soccer, squash, taekwondo, tennis, volleyball, and water polo for “mixed”; and canoeing, cycling, mountaineering/trail running, mid/long distance swimming, mid/long distance running, and triathlon for “endurance” disciplines*.

* *All daytime recordings included at least one hard training session*.

*Post hoc for pairwise comparisons:*

a *significantly different from skill;*

b *significantly different from power;*

c *significantly different from mixed; and*

d *significantly different from endurance*.

Holter results are shown in Table 2. Sinus bradycardia was the most frequent condition (present in the great majority of athletes during Holter monitoring), with the highest proportion of severe — including both daytime and nighttime — bradycardia in endurance athletes. Two-second pauses were mainly detected during night-time (22 vs. 6% for daytime), with 3-s pauses found only in one endurance athlete (long-distance runner). AV blocks were also overall more frequent during night-time, but no differences were found attending to the different sports disciplines. Third-degree AV blocks (both during day- and night-time) were detected only in one athlete (i.e., complete congenital AV block) whereas high degree AV blocks during night-time were found in seven athletes (a wrestler, a soccer player, three basketball players, a cyclist, and an endurance runner), of whom two (the wrestler and the runner) also showed this condition during daytime. Premature atrial beats were prevalent (62%), with the highest prevalence observed among endurance athletes. AF/flutter, wandering pacemaker, supraventricular tachycardia, and junctional rhythms (premature junctional beats or idioventricular rhythm) were uncommon findings (all <1% except for supraventricular tachycardia [4.0%]). Isolated PVC were found in 39.4% of the athletes undergoing Holter monitoring, ranging between 1 and 99 in 31.2% athletes (with most of them [21%] in the 1–10 range), 100 and 999 in 6.3% and ≥1,000 in 2.0%, with the most frequent morphology (29.7%) being monomorphic PVC (i.e., LBBB [16.0%], RBBB [12.4%], or QS pattern in MCL1, III and CM5 with superior axis [1.2%]), followed by two morphologies (8.4%) and polymorphic morphology (1.4%, with 6 of the 9 cases being endurance athletes). On the other hand, PVC couplets were relatively frequent (10.7%) but complex ventricular arrhythmias were not frequent (PVC triplets: 1.8%; sustained ventricular tachycardia: 0.0%; and nonsustained ventricular tachycardia: 1.5%).

**Table 2:** Cardiac rhythm alterations during Holter monitoring.

	**Overall** ***N*** = **654**	**Skill** ***N*** = **43**	**Power** ***N*** = **135**	**Mixed** ***N*** = **179**	**Endurance** ***N*** = **297**	***p*** **-value**
**Mean daytime rhythm**						
Sinus rhythm	629 (96.2%)	42 (97.7%)	131 (97.0%)	172 (96.1%)	284 (95.6%)	0.853
Sinus bradycardia	625 (95.6%)	37 (86.0%)	126 (93.3%)	173 (96.6%)	289 (97.3%)	0.004
**Daytime bradycardia**						
Mild	136 (20.8%)	17 (39.5%)	39 (28.9%)	39 (21.8%)	41 (13.8%)	<0.001
Moderate	319 (48.8%)	15 (34.9%)	64 (47.4%)	97 (54.2%)	143 (48.1%)	0.135
Severe	164 (25.1%)	5 (11.6%)	22 (16.3%)	34 (19.0%)	103 (34.7%)	<0.001
Extreme	6 (0.9%)	0	1 (0.7%)	3 (1.7%)	2 (0.7%)	-
**Night-time bradycardia**						
Mild	21 (3.2%)	6 (14.0%)	6 (4.4%)	4 (2.2%)	5 (1.7%)	<0.001
Moderate	192 (29.4%)	19 (44.2%)	57 (42.2%)	55 (30.7%)	61 (20.5%)	<0.001
Severe	381 (58.3%)	15 (34.9%)	67 (49.6%)	106 (59.2%)	193 (65.0%)	<0.001
Extreme	56 (8.6%)	2 (4.7%)	4 (3.0%)	14 (7.8%)	36 (12.1%)	0.068
**Atrial rhythms**						
Premature atrial beat	405 (61.9%)	25 (58.1%)	76 (56.3%)	108 (60.3%)	196 (66.0%)	<0.001
AF/flutter	3 (0.5%)	1 (2.3%)	0	2 (1.1%)	0	-
Wandering pacemaker	5 (0.8%)	0	4 (3.0%)	1 (0.6%)	0	0.010
Supraventricular tachycardia	26 (4.0%)	1 (2.3%)	6 (4.4%)	6 (3.4%)	13 (4.4%)	0.875
**Junctional rhythms**						
Premature junctional beat	4 (0.6%)	0	0	0	4 (1.3%)	0.255
Idioventricular rhythm	3 (0.5%)	0	0	2 (1.1%)	1 (0.3%)	-
**Daytime sinus pauses**						
≥2 seconds	42 (6.4%)	1 (2.3%)	5 (3.7%)	10 (5.6%)	22 (7.4%)	0.327
≥3 seconds	1 (0.2%)	0	0	1 (0.6%)	1 (0.3%)	-
**Night-time sinus pauses**						
≥2 seconds	146 (22.3%)	7 (16.3%)	25 (18.5%)	38 (21.2%)	76 (25.6%)	0.264
≥3 seconds	1 (0.2%)	0	0	1 (0.6%)	3 (1.0%)	-
**Daytime AV block**						
1st degree	53 (8.1%)	3 (7.0%)	8 (5.9%)	10 (5.6%)	32 (10.8%)	0.230
2nd degree type I	38 (5.8%)	2 (4.7%)	3 (2.2%)	10 (5.6%)	23 (7.7%)	0.168
2nd degree type II	4 (0.6%)	0	0	1 (0.6%)	3 (1.0%)	-
High degree	2 (0.3%)	0	1 (0.7%)	0	1 (0.3%)	-
3rd degree	1 (0.2%)	0	0	1 (0.6%)	0	-
**Night-time AV block**						
1st degree	85 (13.0%)	7 (16.3%)	11 (8.1%)	22 (12.3%)	45 (15.2%)	0.283
2nd degree type I	95 (14.5%)	5 (11.6%)	10 (7.4%)	29 (16.2%)	51 (17.2%)	0.072
2nd degree type II	22 (3.4%)	1 (2.3%)	1 (0.7%)	9 (5.0%)	11 (3.7%)	0.208
High degree	7 (1.1%)	0	1 (0.7%)	4 (2.2%)	2 (0.7%)	0.341
3rd degree	1 (0.2%)	0	0	1 (0.6%)	0	-
**PVC**						
*Frequency*						
1–99	204 (31.2%)	14 (2.1%)	39 (5.9%)	57 (8.7%)	94 (14%)	0.665
100–999	41 (6.3%)	4 (9.3%)	8 (5.9%)	12 (6.7%)	17 (5.7%)	0.823
≥1,000	13 (2.0%)	2 (4.7%)	3 (2.2%)	4 (2.2%)	4 (1.3%)	0.514
*Morphology*					
LBBB	105 (16%)	10 (23.3%)	22 (16.3%)	27 (15.1%)	46 (15.5%)	0.065
Inferior axis	53 (8.1%)	9 (20.9%)	10 (7.4%)	15 (8.4%)	19 (6.4%)	
Superior axis	50 (7.6%)	1 (2.3%)	11 (8.1%)	12 (6.7%)	26 (8.8%)	
Horizontal axis	2 (0.3%)	0	1 (0.7%)	0	1 (0.3%)	
RBBB	81 (12.4%)	8 (18.6%)	16 (11.9%)	20 (11.2%)	37 (12.5%)	0.832
Inferior axis	43 (6.6%)	3 (7.0%)	7 (5.2%)	14 (7.8%)	19 (6.4%)	
Superior axis	31 (4.7%)	4 (9.3%)	8 (5.9%)	5 (2.8%)	14 (4.7%)	
Horizontal axis	7 (1.1%)	1 (2.3%)	1 (0.7%)	1 (0.6%)	4 (1.3%)	
QS pattern in MCL1, III, and CM5 with superior axis	8 (1.2%)	1 (2.3%)	2 (1.5%)	4 (2.2%)	1 (0.3%)	0.261
Two morphologies	55 (8.4%)	1 (2.3%)	9 (6.7%)	20 (11.2%)	25 (8.4%)	0.884
LBBB + RBBB	48 (7.3%)	1 (2.3%)	8 (5.9%)	18 (10.1%)	21 (7.1%)	
LBBB + QS pattern	7 (1.1%)	0	1 (0.7%)	2 (1.1%)	4 (1.3%)	
Polymorphic	9 (1.4%)	0	1 (0.7%)	2 (1.1%)	6 (2.0%)	0.908

*Data are N (%). No cases were found of Brugada syndrome, neither of short or long QT. Eleven athletes had Wolf-Parkinson-White, none of whom showed reentry tachycardia (neither orthodromic or antidromic). AF, atrial fibrillation; AV, atrioventricular; LBBB, left bundle branch block; PVC, premature ventricular contraction; RBBB, right bundle branch block. Significant p-values for group (i.e., type of sport) effect are in bold. Details for degrees of bradycardia: mild, 59–50 bpm; moderate, 49–40 bpm; severe, 39–30 bpm; extreme, ≤29 bpm. The bold values indicate the significant p-values (< 0.05)*.

Echocardiographic findings in the athletes that underwent Holter monitoring are shown in Table 3. No statistical associations were found between arrhythmias (including the different PVC morphologies) and echocardiography-determined variables and cardiac structural conditions (including mitral valve prolapse) (all p > 0.1). On the other hand, an association was found between mild mitral regurgitation and supraventricular (odds ratio [OR] 2.61; 95% confidence interval (CI), 1.08 to 6.32; *p* = 0.03) and ventricular (OR 2.80; 95% CI, 1.15 to 6.78; *p* = 0.02) arrhythmias, as well as between mild or moderate mitral regurgitation and ventricular arrhythmias (OR 2.49; 95% CI, 1.03 to 6.01; *p* = 0.04). A trend (*p* = 0.06) was also found for an association between mild or moderate mitral regurgitation and supraventricular arrhythmias (OR 2.12; 95% CI, 0.98 to 5.65).

**Table 3:** Echocardiography findings in elite athletes that underwent Holter monitoring (*n* = 654).

**Valvular regurgitations**	
Mild aortic regurgitation	21
Moderate aortic regurgitation	9
Severe aortic regurgitation	0
Mild pulmonary regurgitation	124
Moderate pulmonary regurgitation	19
Severe pulmonary regurgitation	0
Mild mitral regurgitation	165
Moderate mitral regurgitation	14
Severe mitral regurgitation	1
Mild tricuspid regurgitation	218
Moderate tricuspid regurgitation	15
Severe tricuspid regurgitation	0
Cardiomyopathies^*^	
Hypertrophic cardiomyopathy	6
Dilated cardiomyopathy	2
Arrhythmogenic cardiomyopathy	2
LV non-compaction cardiomyopathy	8
Ischemic cardiomyopathy	1
**Congenital heart diseases**	
Mitral prolapse^**^	69
Ventricular septal defect	2
Atrial septal defect	1
Patent *foramen ovale*	6
Partial mitral clef	1
Atrial septal aneurysm	4
Tricuspid prolapse	1
Aortic disease	7
Bicuspid aortic valve	7
Idiopathic dilatation of pulmonary artery	2
Anomalous systemic venous drainage	1
Ebstein anomaly	1
LV myocardial cleft	1
Atrial myxoma	1

*Data are N (%)*.

*LV, left ventricular*.

*All the aforementioned conditions were diagnosed through echocardiographic evaluation by the same experienced cardiologist (A.B.)*

* *All cardiomyopathies were corroborated with cardiac resonance imaging*.

** *No case of mitral annular disjunction was found. The severity (mild, moderate, or severe) of valve regurgitations was determined following the recommendations of the Spanish society of cardiac imaging (https://ecocardio.com/documentos/valores-referencia/371-criterios-de-cuantificacion-de-valvulopatias.html)*.

## Discussion

The main finding of our study was the low proportion of dangerous cardiac arrhythmias (e.g., PVC triplets, nonsustained or sustained ventricular tachycardia, polymorphic PVC, high-degree atrioventricular block or idioventricular rhythm) irrespective of the sports discipline, in young elite athletes who required Holter monitoring due to the presence of symptoms or abnormal echocardiographic/ECG findings. Also, the presence of arrhythmias or the detection of ≥1,000 PVC during Holter monitoring was not associated with underlying major structural alterations. Furthermore, with the exception of bradycardia no overall differences were found in Holter findings attending to the main type of sports discipline. Participation in endurance sports was not associated with a higher risk of major cardiac rhythm alterations compared to the other types of sports, including those with a comparatively low cardiovascular demand (i.e., “skill” sports such as shooting events).

Most elite athletes (96%) undergoing Holter evaluation—and particularly endurance athletes during night-time (i.e., 99% of them)—presented with sinus bradycardia, thereby reflecting the well-documented exercise (and mainly endurance) training-induced vagotonic effect with a decrease in the intrinsic firing rate of the sinoatrial node (1). Sports-associated bradycardia therefore seemed to represent an essentially physiological “healthy” adaptation, at least in our large population sample. In fact, extreme bradycardia (<30 bpm) was an uncommon finding during daytime (<1%) and infrequently identified relatively at night (9%), and was not related to any underlying cardiac condition. Sinus pauses lasting ≥3 s and 2nd degree type II and 3rd degree AV blocks were also infrequent (<1%). In line with our findings, previous studies have reported a very high prevalence of sinus bradycardia (up to 94%) among athletes and a very low prevalence of extreme bradycardia, with the latter not related to any underlying cardiac condition (22, 23). Our findings are also in agreement with a study in 120 competitive athletes in whom only 14 presented with ≥3-s pauses, and no deaths were registered during a mean follow-up of 7.5 years (24). The authors concluded that the presence of ≥3-s pauses should not exclude sport participation, which is also supported by the present findings. High-grade AV blocks were also rarely identified in the present study (<1%), which is in agreement with previous reports (25, 26) except for Viitsalo et al. (27), who described the presence of AV blocks in 20% of 37 young athletes followed by Holter monitoring.

AF/flutter was an uncommon finding among the elite athletes who underwent Holter monitoring in the present study (3 of 654 athletes), which is in line with our previously published findings (11) and with the results reported in a similar cohort of Italian athletes (28). There is, however, conflictive data regarding the effects of regular exercise on the development of AF. For instance, there is evidence that regular leisure-time physical activity is not associated with the prevalence of AF and can even reduce the risk of this condition (29, 30), which is in accord with the overall antiarrhythmogenic effect of regular exercise at least at the non-elite level (4). Yet, sports participation seems to be associated with a higher prevalence of AF (29, 31). In particular, strenuous endurance exercise might trigger the occurrence of arrhythmias (predominantly, but not only, AF) through several mechanisms such as myocardial fibrosis and inflammation (32). Aagaard et al. (33) recently reported a higher prevalence of AF in former professional football players than in the general population, which was associated with slowed cardiac conduction. Andersen et al. (5) assessed 52,755 long-distance cross-country skiers and observed a higher incidence of AF among those who were faster or completed more races. The potential causes for AF occurrence, especially in young athletes, remains to be determined, although atrial remodeling toward “sphericity” (*i.e*., enlargement mainly in the horizontal axis) might be involved (11). In any case, AF is a condition associated with age, including in elite athletes (11), which decreases the likelihood of encountering this condition in a cohort of young elite athletes such as that assessed here.

The prevalence of premature atrial and ventricular beats has been commonly reported as 40–90% in the general population (34, 35) and 6–70% in athletes (36). In the present study, premature atrial and ventricular beats were quite common (61.9% and 39.4%, respectively) among elite athletes who underwent Holter monitoring, although these figures represent a small percentage of the entire sample (6.2 and 3.9%, respectively). By comparison, Ben Halima et al. (8) reported the presence of PVC in resting ECG in 42 of 5,789 athletes, with three cases of hypertrophic cardiomyopathy, one of arrhythmogenic cardiomyopathy, one of compression of the right ventricle due to pectus excavatum, and two of ventricular tachycardia episodes, and with five athletes excluded from sports participation. Also, Biffi et al. (9) reported the presence of PVC in 355 (2.2%) of 15,899 competitive athletes. Cardiac pathology was detected in 7% of cases and one death was described due to arrhythmogenic cardiomyopathy while training against medical advice. In the present study, mitral valve prolapse was the most frequent congenital heart disease among those athletes undergoing Holter monitoring. Similarly, Biffi et al. (9) reported mitral valve prolapse as the most common cardiac abnormality in athletes, with this condition associated with the number of PVC. In our study, however, we found no association between any of the analyzed arrhythmias (including the dangerous arrhythmias) and echocardiographic findings other than mild or moderate mitral regurgitation. Further research is needed to determine the potential mechanisms linking mitral regurgitation and arrhythmias in elite athletes. In this regard, intense exercise training induces a significant dilation of annular dimensions and increases leaflet tenting of the mitral valve, potentially leading to mild mitral regurgitation (37). Indeed, mild mitral regurgitation was frequent among those athletes undergoing Holter evaluation (i.e., present in one-fourth of them). Our findings suggest the need for close monitoring of those cases with mild or moderate mitral regurgitation, as this condition might reflect an “insufficient” geometrical adaptation of the mitral annulus to regular, intense exercise (37). On the other hand, the prevalence of mitral regurgitation in those athletes undergoing Holter evaluation is overall comparable to that reported for young non-athletic populations (e.g., 24% in people aged 20 to 39 years) (38).

The main limitations of the present study are its retrospective design with no subsequent follow-up, the young age of most elite athletes (<30 years on average, albeit with a mean 10-year competition experience), which precludes making potential inferences on potential sequelae of sports participation in the longer term, and the Holter analysis of only part (~10%) of the total sample (i.e., those with family history, reporting cardiovascular symptoms, with suspicion of cardiac structural abnormalities potentially associated with dangerous arrhythmias, or with ECG features prompting a closer examination). Thus, while the remainder of athletes showed normal ECG and cardiovascular results (including echocardiographic evaluations, with a mean of three assessments per athlete) and no self-reported symptoms over the years, we might still have missed the occurrence of some cases of arrhythmias (i.e., silent cases or athletes unwilling to recognize cardiovascular symptoms). In addition, it is quite possible that athletes competing in a national team had been previously screened in their local/regional team, which minimizes the risk of reaching the elite competition level with an undiagnosed major structural or electrical cardiac abnormality. Moreover, it is unlikely that those individuals with severe structural or arrhythmic diseases could reach a very high, elite performance level. On the other hand, the use of cardiac magnetic resonance in all the athletes undergoing Holter monitorization [at least in those showing ventricular arrhythmias, as done in recent research (12, 13, 15, 16)] might have allowed to perform accurate myocardial tissue characterization and a more precise diagnosis of some cardiac abnormalities, as opposed to the echocardiographic evaluation we conducted. Notably, isolated nonischemic left-ventricular (LV) late gadolinium enhancement with a stria pattern may be associated with life-threatening arrhythmias and sudden death in the athlete (12). In this effect, LV scar is often not detected by echocardiography owing to its subepicardial/midmyocardial location (12). In addition, we did not perform strain analysis because this technique was not available in our center during the start of the study period. In turn, the major strengths of our study are the large sample size analyzed (which to our knowledge is the largest elite athlete population evaluated by Holter monitoring so far), the inclusion of different types of sports disciplines (including specialties with a comparatively low cardiovascular demand vs. those with the highest demands) as well as of female athletes, and the comprehensive cardiac evaluation that all elite athletes underwent.

In conclusion, our results suggest that arrhythmias, especially dangerous ones (e.g., nonsustained ventricular tachycardia, frequently polymorphic PVC, high-degree atrioventricular block) are infrequent among young elite athletes, as detected with Holter monitoring due to the presence of symptoms, suspicious echocardiographic alterations, or abnormal ECG findings. Furthermore, the existence of cardiac structural conditions does not appear to increase the risk for cardiac rhythm alterations. Nonetheless, it would seem prudent to closely monitor the athlete in the event of mitral regurgitation.

## Data availability statement

The raw data supporting the conclusions of this article will be made available by the authors, without undue reservation.

## Ethics statement

The studies involving human participants were reviewed and approved by Universidad Europea Miguel de Cervantes. The patients/participants provided their written informed consent to participate in this study.

## Author contributions

Full access to all the data in the study and take responsibility for the integrity of the data and the accuracy of the data analysis: AB and M-EH. Concept, design, and supervision: AB and AL. Acquisition of data: AB, M-EH, FM-A, LD-G, MA-A, and SB-M. Data analysis: FM-A and AS-L. Drafting of the manuscript: AB, FM-A, PV, LD-G, AS-L, and AL. Interpretation of data and critical revision of the manuscript for important intellectual content: All authors. All authors contributed to the article and approved the submitted version.

## Funding

Research by PV was funded by a postdoctoral contract granted by Instituto de Salud Carlos III (Sara Borrell grant, CD21/00138). Research by AL was funded by the Spanish Ministry of Economy and Competitiveness and Fondos Feder [AL, grant PI18/00139].

## Conflict of interest

The authors declare that the research was conducted in the absence of any commercial or financial relationships that could be construed as a potential conflict of interest.

## Publisher's note

All claims expressed in this article are solely those of the authors and do not necessarily represent those of their affiliated organizations, or those of the publisher, the editors and the reviewers. Any product that may be evaluated in this article, or claim that may be made by its manufacturer, is not guaranteed or endorsed by the publisher.
