# Supplementary material for: Holter-determined arrhythmias in young elite athletes with suspected risk: Insights from a 20-year experience
Source: Front Cardiovasc Med. 2022 Jul 22;9:896148. doi: 10.3389/fcvm.2022.896148 (PMC9354520; doi:10.3389/fcvm.2022.896148)
Supplement: Supplementary file 1 [file Table_1.DOCX]

**Supplementary file 1.** Demographic, sports and training characteristics of the participants who underwent Holter monitoring or not, respectively.

|  | **Athletes not undergoing Holter evaluation**  ***N* = 5925** | | **Athletes undergoing Holter evaluation**  ***N* = 654** | ***P*-value** |
| --- | --- | --- | --- | --- |
| **Proportion of women** | 35% | | 28% | <0.001 |
| **Type of sports** |  | |  | <0.001 |
| Skill | 8% | | 7% |  |
| Power | 27% | | 21% |  |
| Mixed | 29% | | 27% |  |
| Endurance | 36% | | 45% |  |
| **Age (years)** | 24 (19-26) | | 24 (19-28) | <0.001 |
| **Training regimen (hours/week)** | 19 ± 8 | | 19 ± 9 | 0.163 |
| **Years in competition** | 10 ± 6 | | 8 ± 6 | <0.001 |
| **VO_2_max (ml/kg/min)** | 54 ± 9 | | 57 ± 10 | <0.001 |
| **Resting ECG heart rate (bpm)** | 56 ± 13 | | 61 ± 11 | <0.001 |
| **Main echocardiographic features** | | |  |  |
| LVEDD (mm) | 52.4 ± 5.2 | | 54.4 ± 6.2 | 0.004 |
| LVESD (mm) | 35.1 ± 7.4 | | 36.4 ± 4.5 | 0.187 |
| IVSd | 8.6 ± 1.4 | | 9.3 ± 1.4 | <0.001 |
| PWT (mm) | 8.4 ± 1.3 | | 9.2 ± 1.3 | <0.001 |
| RVEDD (mm) | 30.2 ± 6.0 | | 30.1 ± 5.7 | 0.971 |
| LAD (anteroposterior, mm) | 34.0 ± 5.1 | | 36.2 ± 5.8 | 0.001 |
| LAD (superoinferior, (mm) | 50.3 ± 6.5 | | 53.4± 6.4 | <0.001 |
| RAD (superoinferior, mm) | 52.3 ± 6.1 | | 54.1 ± 6.2 | <0.001 |
| LV mass (g) | 162.5 ± 50.6 | | 194.4 ± 58.6 | <0.001 |
| LVEF (%) | | 60.9 ± 7.1 | 60.5 ± 7.3 | 0.653 |
| LVFS (%) | | 0.33 ± 0.11 | 0.33 ± 0.05 | 0.981 |
| E/A | | 2.1 ± 0.7 | 2.2 ± 1.1 | 0.331 |

Data are % or mean ± SD. Abbreviations: *A*, late ventricular filling wave; *E*, early ventricular filling wave; IVSd, interventricular septal wall thickness at end-diastole; LAD, left atrial dimension; LVEF, left ventricular ejection fraction; LVEDD, LV end-diastolic diameter; LVESD, LV end-systolic diameter; LVFS, LV fractional shortening; RAD, right atrial dimension; RVEDD, right ventricle end-diastolic diameter at outflow tract in M-mode; PWT, posterior wall thickness at end-diastole; VO_2_max, maximum oxygen uptake.
